# Kirschner Wire Temporary Intramedullary Fixation Combined with a Locking Anatomical Plate versus a Reconstruction Plate in the Treatment of Comminuted Clavicular Fractures: A Retrospective Study

**DOI:** 10.1155/2018/5017162

**Published:** 2018-12-23

**Authors:** Jianxin Xie, Danfeng Xu, Xiaofei Zheng, Mingdi Zhou, Wei Ouyang, Tao Zhang, Lei Lu

**Affiliations:** Department of Orthopaedic Surgery, Shaoxing Central Hospital, Hua-yu Road 1, Keqiao, Shaoxing, 312000, China

## Abstract

We investigate the clinical efficacy of Kirschner wire temporary intramedullary fixation combined with a locking anatomical plate for the treatment of comminuted clavicular fractures. We retrospectively studied 112 patients [80 (71%) men] treated between February 2007 and February 2014. The patients were allocated to treatment with Kirschner wire temporary intramedullary fixation combined with a locking anatomical plate [minimally invasive group (G_M_)] or a reconstruction plate [traditional group (G_T_)]. The 112 patients were followed up for 12–48 months (mean, 14 months). The operation time was significantly shorter in G_M_ than in G_T_. Intraoperative blood loss was significantly less in G_M_ than in G_T_. The total incision length was significantly shorter and the visual analog scale pain score 24 h after surgery was significantly lower in G_M_ than in G_T_. Fracture healing time was significantly shorter and the complication rate was significantly lower in G_M_ than in G_T_. No significant difference in shoulder function score was observed between groups. We recommend Kirschner wire temporary intramedullary fixation combined with a locking anatomical plate as the treatment of choice for comminuted clavicular fractures because of the shorter operation time, lesser intraoperative blood loss, easier reduction of the operation, quicker fracture healing, and lower postoperative complication rate.

## 1. Introduction

Fractures of the clavicle, 80% of which are located midshaft, account for 12% of all bone fractures. Approximately 19% of midshaft clavicular fractures are comminuted [1–3]. Comminuted fractures are usually high-energy fractures. They are not as stable as two-segment fractures, and risk of symptomatic nonunion is greater due to the presence of soft-tissue injury [4]. Traditionally, displaced midshaft clavicle fractures have been managed by nonoperative treatment, even in cases with comminuted displacement [2, 4, 5]. However, nonoperative treatment has been shown to result in higher levels of nonunion and malunions, as well as poorer functional recovery [6, 7]. Recently, management of comminuted clavicular fractures has shifted to surgical intervention and surgical options, such as plate fixation or intramedullary fixation [8–10].

Extensive periosteal stripping is required for internal fixation with a traditional reconstruction plate, and some patients develop complications, such as fracture of the internal fixation material or refracture after removal of the internal fixation device during follow-up. We began to treat comminuted clavicular fractures with open reduction and the use of a Kirschner wire for temporary intramedullary fixation combined with a reconstruction plate in September 2006. This method does not require extensive periosteal dissection during internal fixation and causes little trauma to surrounding soft tissues, blood vessels, and nerves. Hence, we conducted a retrospective study comparing Kirschner wire temporary intramedullary fixation combined with a locking anatomical plate with the same fixation combined with a reconstruction plate for the treatment of comminuted clavicular fractures. Our null hypothesis was that Kirschner wire temporary intramedullary fixation combined with a locking anatomical plate would be a more efficacious and safer than the traditional method.

## 2. Methods

### 2.1. Patients

Ethical approval was obtained from the Ethics Committee of Shaoxing Central Hospital. All enrolled patients gave informed consent. In total, 112 patients with comminuted clavicular fractures treated between February 2007 and February 2014 were enrolled in this retrospective study. The inclusion criteria were completely displaced fracture fragments (displacement > 2 cm), shortening > 2 cm, segmental fractures, associated neurovascular injury, and patients requiring rapid return of function. Exclusion criteria were open or pathological fractures, bilateral clavicle fractures, infective or concomitant disease, and previous surgeries on the affected shoulder [11].

The patients were preoperatively informed of the advantages and disadvantages of both surgical procedures, costs of internal fixation materials, and potential complications. The surgical procedure was selected based on patient willingness and financial capacity. Ultimately, 52 cases received Kirchner wire temporary intramedullary fixation combined with a locking anatomical plate as a minimally invasive treatment for comminuted clavicular fractures [minimally invasive group (G_M_)]. Additionally, 60 cases received the same fixation with a traditional reconstruction plate [traditional group (G_T_)]. Baseline patient data are provided in Table 1.

### 2.2. Surgical Method

The first author and another senior orthopedic surgeon performed all surgeries.

#### 2.2.1. Minimally Invasive Group

**Figure 1:**
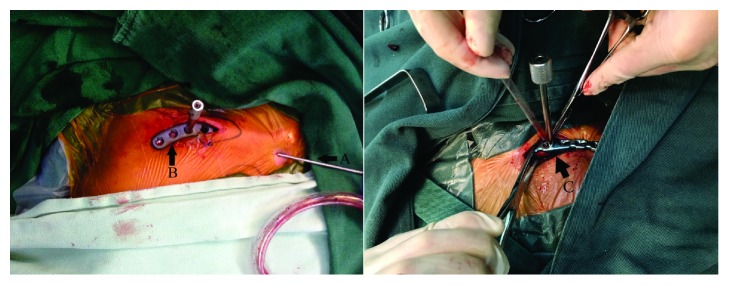
A: after fracture reduction, the temporary intramedullary fixation of Kirschner wire maintains the stability of the fracture, and B: the anatomic locking plate is inserted through the incision. C: extensive periosteal dissection during internal fixation when using reduction clamp.

**Figure 2:**
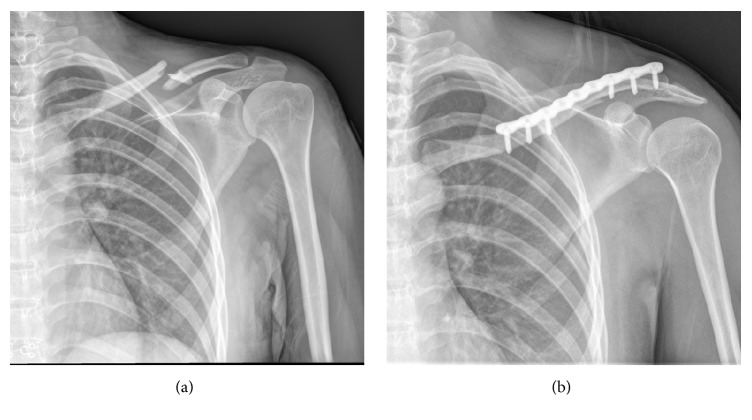
(a) Left closed comminuted midshaft clavicle fracture; (b) fracture end anatomical reduction could be seen in X-ray films after minimally invasive Kirchner wire temporary intramedullary fixation combined locking anatomical plate.

Each patient was given an intravenous injection of antibiotics 30 min prior to surgery. Patients with poor cardiopulmonary function, but normal coagulation function (*n* = 15), received cervical plexus block anesthesia and those with good cardiopulmonary function, but abnormal coagulation function (*n* = 37), received general anesthesia. Each patient was placed in the supine position, the affected shoulder was raised, and the head was turned to the healthy side. After preparing the skin, a 3 cm long incision centered on the fractured end was made, the fractured end was exposed, and a large Kirchner wire (diameter 1.5–2.5 mm, adjusted according to medullary cavity size) was placed from the lateral medullary cavity of the fractured end using an electric drill and the retrograde needle-threading method. The Kirschner wire penetrated the bone cortex and was pulled out of the skin, so that its proximal portion was parallel to the distal fractured end. After reducing the fracture, the Kirschner wire was threaded retrogradely into the medullary cavity from the lateral side, which was not further drilled after the contralateral cortex had been penetrated. In this manner, Kirschner wire temporary intramedullary fixation was accomplished successfully. The fractured end was observed under a C-arm X-ray device to guarantee anatomical reduction. Subsequently, a locking anatomical plate of an appropriate length was placed in the incision along the periosteum, and incisions about 0.3 cm in length were made according to the surface projections of the acromial end of the locking plate and the locking screw at the internal clavicle end (Figure 1). The locking hole sleeves were screwed into the acromial and internal ends of the clavicle, the holes were drilled, and locking screws of an appropriate length were screwed in after measuring the depth. The exposed Kirchner wire was pulled out about 1 cm, and a locking screw of an appropriate length was screwed in percutaneously until the screw did not touch the Kirchner wire. Similarly, the Kirchner wire was removed, as for the percutaneous screw-in of the locking screw, until removal of the Kirchner wire and the screw-in of the last locking screw. The fractured end was observed under a C-arm X-ray device to guarantee anatomical reduction. Then, the incisions were washed and sutured. Typical cases are shown in Figure 2.

#### 2.2.2. Traditional Group

**Figure 3:**
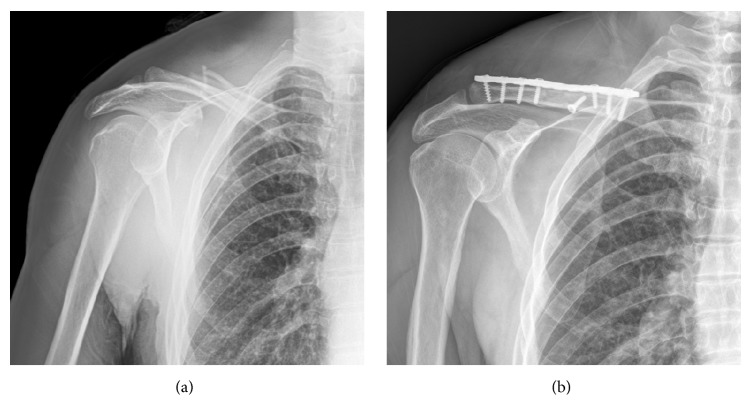
(a) Right closed comminuted midshaft clavicle fracture; (b) fracture end anatomical reduction could be seen in X-ray films after extensive incision traditional reconstruction plate internal fixation.

Each patient was given an intravenous injection of antibiotics 30 min prior to surgery. Patients with poor cardiopulmonary function, but normal coagulation function (*n* = 12), received cervical plexus block anesthesia, and those with good cardiopulmonary function, but abnormal coagulation function (*n* = 48), received general anesthesia. Each patient was placed in the supine position, the affected shoulder was raised, and the head was turned to the healthy side. After preparing the skin, an 8–12 cm long incision centered on the fractured end was made. The fractured end was exposed, the fractured end clots were eliminated, and the fractured end periosteum was stripped. An ordinary screw was occasionally used to fix the fractured end, based on the size of the fractured fragment, so that the reduction could be maintained. A 3.5 mm reconstruction plate of appropriate length was shaped, followed by measurement of the depth and screw-in of a screw of appropriate length. The fractured end was observed under a C-arm X-ray device to guarantee anatomical reduction. Then, the incisions were washed and sutured. Typical cases are shown in Figure 3.

### 2.3. Postoperative Management

Each patient received an intravenous injection of antibiotics after surgery. Pendular functional exercise of the affected limb was encouraged on the second day after surgery, and elevation and abduction of the shoulder joint were encouraged at 3 weeks after surgery.

### 2.4. Observational Indices

The operation time was calculated from the beginning of skin incision to completion of skin sutures. The total incision length was measured using a steel scale after the incision was sutured. Blood loss was calculated based on the intraoperative negative pressure suction amount + gauze wiping amount [weight of all pieces of wiped gauze after surgery − weight of all pieces of wiped gauze before surgery, which was converted according to 1 g (blood weight) = 1 ml (blood volume)]. The pain score at 24 h postoperatively was obtained using a visual analog scale (VAS) [12]. The fracture healing time was determined from reexamination of X-ray films every 2–3 weeks after discharge until complete bone union was observed. Shoulder function was evaluated using the Neer shoulder joint assessment scale [13] at 3 months after surgery. Postoperative complications included postoperative incision infection, incision malunion, internal fixation material fracture, and refracture.

### 2.5. Statistical Analysis

Data were analyzed with SPSS 22.0 statistical software (IBM Corp., Armonk, NY, USA). Continuous variables are expressed as means ± standard deviations and were analyzed using the independent-samples* t* test. Enumerated data were analyzed using the chi-squared test.* P *values < 0.05 were considered to be significant.

## 3. Results

All fractures were fixed successfully. The operation time, intraoperative blood loss, total incision length, 24 h postoperative VAS score, and fracture healing time were superior in G_M_ than in G_T_. The shoulder function score at 3 months after surgery did not differ between groups. No complication was observed in G_M_. Two cases in G_T_ had incision malunion, which recovered after dressing change. Another two cases had fractures of the internal fixation material, which healed after removal or replacement of the material and removal of the autologous iliac crest graft. In addition, one case developed refracture after the removal of internal fixation, which healed after conservative treatment. The incidence of complications was much lower in G_M_ than in G_T_ (Table 2). The 112 patients were followed up for 12–48 months (mean, 14 months), and bone union was achieved in all cases.

## 4. Discussion

This study evaluated two different surgical procedures for comminuted clavicular fractures, with particular attention to any possible differences in terms of intraoperative details, fracture healing time, functional recovery, and complications. There were significant differences in operation time, intraoperative blood loss, incision length, healing time, and complication rates between the two procedures. Use of Kirschner wire temporary intramedullary fixation combined with a locking anatomical plate showed better results than use of a reconstruction plate.

Kirschner wire fixation is a traditional method of open reduction and internal fixation of clavicle fractures. However, with Kirschner wire fixation, it is difficult to achieve good fixation in each force and motion plane [14]. Kirschner wire loosening, slippage into the lung [15], and injury of the brachial plexus have also been reported [16]. The traditional reconstruction plate method use in treatment of clavicular fractures frequently requires an extensive incision, with a reconstruction plate used to strengthen the internal fixation after stripping of the periosteum, which is associated with large surgical wounds and intraoperative blood loss. In addition, stripping of the periosteum around the fractured end increases the probability of fracture nonunion. The importance of protecting the soft tissue has been increasingly recognized with the promotion and application of locking plate use and a minimally invasive concept.

An incision of about 3 cm in length, centered on the fractured end, was made in G_M_. Kirchner wire was used for temporary intramedullary fixation of the midshaft clavicle, the fractured end line of force was restored and maintained, and repeated reduction of the fracture end was not necessary. Subsequently, the locking anatomical plate was inserted, and locking screws of appropriate length were screwed in percutaneously. This approach is associated with a smaller surgical wound. An extensive skin incision is required to expose the fractured end and strip the periosteum when using a traditional reconstruction plate to treat a comminuted clavicular fracture. The fractured end must be repeatedly reduced intraoperatively, which may lead to accidental injury of the subclavian nerve or vascular reconstruction plate. Molding is required after fracture reduction and can be placed above the clavicle. Consequently, the Kirchner wire temporary intramedullary fixation surgical procedure combined with a locking anatomical plate for internal fixation was markedly superior to the traditional incision + reconstruction plate internal fixation.

The operation time was shorter in G_M_ than in G_T_. Repeated reduction of the fractured end or molding of the plate was not required, the self-tapping self-drilling screws could be screwed in directly, and the shorter skin sutures effectively shortened the operation. In contrast, more time was spent reducing the fractured end, molding the reconstruction plate, tapping the screws and drilling, and suturing the long incision in the G_T_.

Patients in G_M_ had smaller surgical wounds, less intraoperative blood loss, milder postoperative pain, and shorter fracture healing times than did those in G_T_. In addition, no extensive stripping of the periosteum was required. However, the locking plate is more expensive than an ordinary reconstruction plate and thus has restricted application in some patients with limited finances.

Our follow-up results indicate that union of the comminuted clavicular fractures was achieved in both groups. Moreover, the incidence of postoperative complications was lower in G_M_, and no fracture nonunion or delayed union was observed, which was related to the reduced intraoperative periosteal stripping and the strong internal fixation provided by the locking plate. Importantly, the shorter surgical incision was more easily accepted by patients. In contrast, plate molding is required during fixation with a traditional reconstruction plate, and extensive periosteal stripping is required at the fractured end before internal fixation. This procedure is associated with greater incidence of nonunion of the fractured end, delayed union, and fracture of the internal fixation material, as well as a longer fracture healing time. The longer surgical scar affects aesthetics. Both the locking and reconstruction plates effectively fix the fractured end and allow for early functional exercise of the affected shoulder. No difference in the Neer shoulder score was detected between groups at 3 months after surgery.

The present study had several limitations. First, this was a retrospective study performed in a single center without treatment randomization. Furthermore, the follow-up period was different between patients, and shoulder function was only evaluated 3 months after surgery. Further studies are required to evaluate shoulder function at different stages after the operation. Moreover, the study population was small. Future studies with larger sample sizes are therefore required.

## 5. Conclusion

Because of the shorter operation time, lesser intraoperative blood loss, easier reduction during the operation, quicker fracture healing, and lower postoperative complication rate observed in this study, we suggest that Kirschner wire temporary intramedullary fixation combined with a locking anatomical plate is a safe and effective minimally invasive surgical treatment for comminuted clavicular fractures.

## Figures and Tables

**Table 1:** Baseline data of all patients.

	G_M_ (n=52)	G_T_ (*n*=60)	*T or χ* ^2^ value, *P-value*
Mean age in years	46.3±3.7	45.7±4.1	*t*=0.81, 0.421
Sex (male/ female)	40/12	40/20	*x* ^2^=1.44, 0.231
Course of disease (Day)	6.6±0.3	6.4±0.4	*t*=2.95, 0.044
Fracture side (Left/ right)	12/40	18/42	*x* ^2^=0.68, 0.409
Cause of injury			*x* ^2^=1.01, 0.601
Traffic injury	35	39	
Falling injury	10	9	
Other injury	7	12	

**Table 2:** Operative details of all patients.

	G_M_ (n=52)	G_T_ (*n*=60)	*T *value, *P-value*
Operation time (min)	45.0±7.5	62.0±15.5	-7.20, 0.000
Intraoperative blood loss (ml)	25.0±16.5	63.0±19.7	-10.96, 0.000
Total incision length (cm)	6.4±0.8	10.3±1.7	-15.14, 0.000
^a^VAS pain score 24h after surgery (points)	1.9±0.5	4.4±0.4	-29.37, 0.000
Fracture healing time (weeks)	9.3±1.2	11.4±1.3	-8.834, 0.000
Incidence of complication	0	8.3% (5/60)	*p*=0.041^*∗*^
^b^Shoulder Neer score3 months after surgery	97.8±1.4	98.1±1.2	-1.22, 0.225

^*∗*^Fisher's test.

## Data Availability

The data used to support the findings of this study are included within the article.
